# Hydrogenation Reactions with Heterobimetallic Complexes

**DOI:** 10.1002/anie.202416100

**Published:** 2024-11-07

**Authors:** Preshit C. Abhyankar, Christine M. Thomas

**Affiliations:** ^1^ Department of Chemistry and Biochemistry The Ohio State University 100 W. 18th Ave. 3109 Newman and Wolfrom Columbus Ohio 43210 United States

**Keywords:** hydrogenation, catalysis, heterobimetallic, metal-metal cooperativity

## Abstract

Hydrogenations are fundamentally and industrially important reactions that are atom economical paths to synthesize value‐added products from feedstock chemicals. The cooperative effects of two or more metal centers in multimetallic active sites is a successful strategy to activate small molecules and facilitate catalytic reactions, and this strategy has been recently applied to catalytic hydrogenation reactions. Furthermore, heterobimetallic complexes have been well‐documented to provide novel reaction pathways and improved selectivity, compared to their homo‐bimetallic and monometallic analogues. This minireview provides a historical perspective on the development of heterobimetallic catalysts for the hydrogenation of unsaturated substrates and describes recent developments in this burgeoning research area.

## Graphical abstract

This minireview describes the development of heterobimetallic homogeneous catalysts for the hydrogenation of unsaturated substrates including alkenes, alkynes, and ketones. These examples highlight the benefits of metal‐metal cooperativity, which can arise from the activation of molecular hydrogen across two different metal centers, bimetallic product elimination steps, and the tuning of electronic properties through metal‐metal communication.

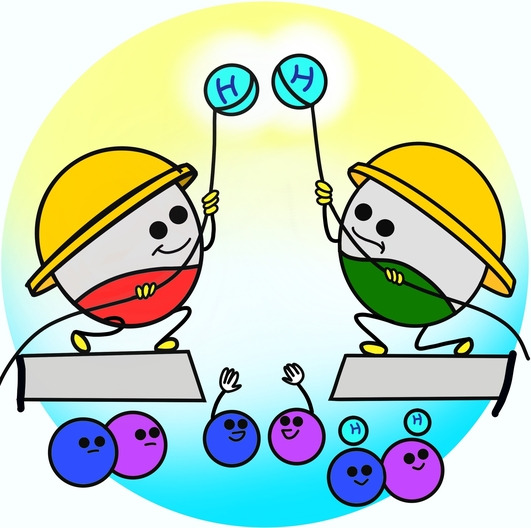

## Introduction

Hydrogenation reactions are ideal for the synthesis of fine and bulk chemicals, including pharmaceuticals,[^1^, ^2^] oils,[^3^, ^4^] and fuels, primarily because of the high atom‐economy of these transformations.[^5^, ^6^, ^7^, ^8^] However, the activation of the relatively strong H−H bond in molecular hydrogen (H_2_) is challenging and generally requires a catalyst or promoter. Various strategies including frustrated Lewis pairs,^[9]^ organocatalysis,[^10^, ^11^] and heterogeneous and homogeneous transition metal catalysis,^[12]^ have been fervently investigated for the activation of H_2_ and its subsequent use in hydrogenation reactions. Among these approaches, transition metal catalysis has been especially successful. Consequently, transition metal‐catalyzed hydrogenation reactions have captured the attention of many researchers and have been widely applied on the industrial scale. The success of molecular transition metal catalysts in hydrogenation reactions stems from the ability of electron‐rich metal centers to activate and cleave H_2_ and form reactive metal hydride complexes that are primed for substrate insertion and subsequent reductive elimination processes.

Although the majority of transition metal hydrogenation catalysts are monometallic, there has been rapidly growing interest in bimetallic catalysts,[^13^, ^14^, ^15^, ^16^, ^17^] including heterobimetallic complexes that incorporate two different metals. Heterobimetallic compounds have garnered attention due to their capacity to promote and control the selectivity of novel reactions inaccessible via their monometallic and homo‐bimetallic analogues.[^18^, ^19^, ^20^, ^21^, ^22^, ^23^, ^24^] Among their many applications, heterobimetallic complexes have demonstrated catalytic activity for the hydrogenation of unsaturated substrates, with heterobimetallic constructs providing a number of distinct advantages, including: (1) access to cooperative pathways to activate H_2_, bind substrates, and eliminate newly formed bonds, (2) stabilization of reactive low‐valent metal centers through metal‐metal bonds,[^25^, ^26^] and (3) stabilization of reactive intermediates at one metal center through electronic communication with the second metal either through a direct metal‐metal interaction or through a bridging ligand. While the heterobimetallic core often remains intact throughout a catalytic cycle, there are also examples of successful homogeneous hydrogenation catalysts where catalytic turnover is contingent upon the reversible cleavage of the core into monometallic fragments (see below).

The broad reactivity and catalytic applications of homogeneous and heterogeneous bimetallic and multimetallic complexes and clusters have been extensively reviewed elsewhere.[^13^, ^14^, ^15^, ^16^, ^17^, ^18^, ^19^, ^20^, ^21^, ^22^, ^23^, ^24^, ^27^] This minireview covers catalytic hydrogenation reactions facilitated by heterobimetallic homogeneous catalysts, specifically focusing on the hydrogenation of unsaturated hydrocarbons (alkenes, alkynes) and ketones, with transfer hydrogenation and CO_2_ hydrogenation excluded for brevity. The exploration of heterobimetallic hydrogenation catalysts began in the late 1980s and early 1990s, but was largely abandoned before a resurgence in interest starting in the mid‐2010s. This minireview aims to place recent developments in a historical context by describing the seminal earlier work that provided the initial inspiration and mechanistic information that guided the development of contemporary heterobimetallic catalysts.

## Systems with Direct Metal‐Metal Interactions

In this section, we will focus specifically on bimetallic hydrogenation catalysts linked through direct metal‐metal bonding or interactions. Some of the earliest examples of heterobimetallic hydrogenation catalysts were comprised of two different late transition metals.

**Scheme 1:**
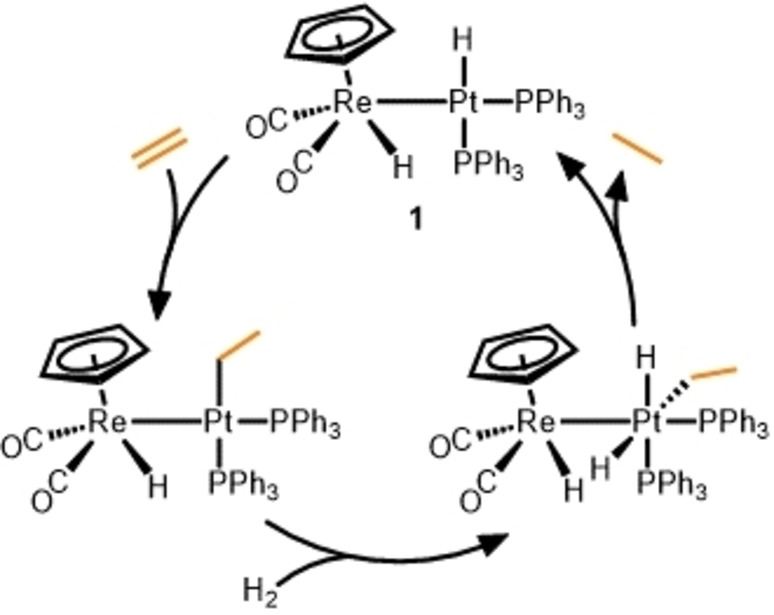
Ethylene hydrogenation using metal‐metal bonded Re/Pt catalyst **1**.^[28]^

In 1989, Casey and co‐workers reported the Re/Pt complex **1**, in which the two metals are only linked through a metal‐metal bond, and demonstrated its ability to catalytically hydrogenate ethylene via a mechanism involving metal‐metal cooperativity (Scheme 1).^[28]^ Although the scope of this catalytic reaction was limited to ethylene and few turnovers were achieved (*TON*=4.2), the bimetallic system was shown to substantially outperform the monometallic Re and Pt analogues, Cp(CO)_2_ReH_2_ and (C_2_H_4_)Pt(PPh_3_)_2_. The proposed mechanism involved ethylene insertion, H_2_ oxidative addition, and reductive elimination occurring at the Pt center, with the Re−Pt bond remaining intact throughout. If this is, indeed, the operative mechanism, the role of the Re center would exclusively be that of a “spectator ligand”, modifying the electronic environment of the Pt center to which it is bound. One could envision, for example, that the Re−Pt bond would decrease the electron density at the Pt center and facilitate the C−H reductive elimination step. In the case of alkyne hydrogenation using **1**, stoichiometric semi‐hydrogenation of the alkyne occurs, generating alkene and alkyne‐bound monometallic Re and Pt complexes, respectively.

**Scheme 2:**
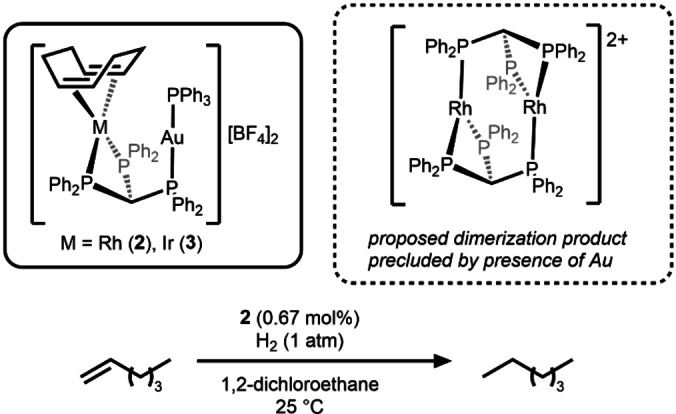
Heterobimetallic M/Au complexes **2**–**3** and the use of **2** as a catalyst for the hydrogenation of 1‐hexene.^[29]^

Osborn et al. reported M/Au (M=Rh (**2**); Ir(**3**)) complexes in 1991 and investigated their catalytic activity for the hydrogenation of 1‐hexene under mild conditions (1 atm, 25 °C) (Scheme 2).^[29]^ Compound **2** was shown to catalytically hydrogenate 1‐hexene more slowly than its monometallic Rh analogue, [Rh(COD){CH(PPh_2_)_3_}][BF_4_] (COD=cyclooctadiene), but with a longer lifetime and improved selectivity. The system shows significant differences in reactivity based on the identity of the group 9 metal; the iridium analogue **3** showed no catalytic activity. Reactions with H_2_ reveal that activation occurs at the Rh or Ir center forming a M(III)‐dihydride species. The Ir−H bonds are thought to be stronger than the Rh−H bonds, contributing to the lower activity of **3**. The authors imply that the role of the Au center may simply be the prevention of deactivation via dimerization, exemplifying the role of the second metal center as a protecting group for highly reactive intermediates.

**Scheme 3:**
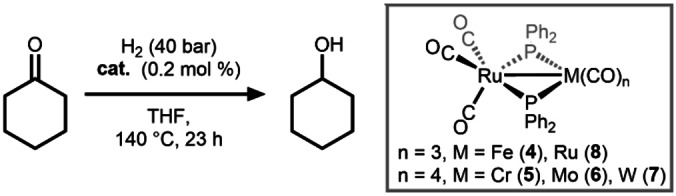
A M/Ru catalyst for ketone hydrogenation.^[30]^ See Table 1 for TON and TOF data.

A series of heterobimetallic Ru/M carbonyl complexes (M=Fe (**4**), Cr (**5**), Mo (**6**), W (**7**), Ru (**8**)) reported in 1992 were evaluated as catalysts for the hydrogenation of cyclohexanone at 140 °C and 40 bar H_2_ (Scheme 3).^[30]^ The importance of tuning the metal combination was adequately illustrated for this platform. Under the conditions outlined in Scheme 3, the homobimetallic Ru_2_ complex **8** (*TON*=205) is significantly more active than the Ru/Fe complex **4** (*TON*=53) and the Ru/Mo compound **6** is a significantly more effective catalyst for cyclohexanone hydrogenation (*TON*=305) (Table 1) than either the Ru/Cr (**5**, *TON*=112) or Ru/W (**7**, *TON*=29) combinations. Thus it appears that the combination of Ru with another 4d metal is optimal, which could be attributed to optimal tuning of M‐hydride bond strengths/reactivity in key intermediates.

**Table 1:** *TON* for cyclohexanone reduction using phosphido‐bridged heterobimetallic compounds **4**–**8** under the conditions outlined in Scheme 3.^[30]^

Catalyst	Yield (%)	TON	TOF (h^−1^)
**4**	10	53	1.9
**5**	21	112	4.9
**6**	56	305	13.3
**7**	6	29	1.3
**8**	41	205	9

**Scheme 4:**
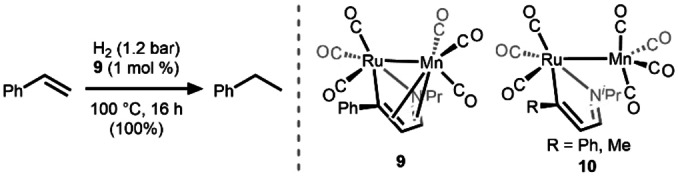
Heterobimetallic Rh/Mn compounds **9** and **10** and hydrogenation of styrene to ethylbenzene using **9**.^[31]^

In 1993, a Ru/Mn complex (**9**) with a bridging monoazadienyl ligand was reported by Elsevier and co‐workers to reduce styrene to ethylbenzene at 100 °C under H_2_ (1.2 atm) (Scheme 4).^[31]^ The reaction was hypothesized to proceed through the coordination of styrene to the Mn center, followed by oxidative addition of H_2_ at Mn. Changes in the hapticity of the monoazadienyl ligand were proposed to stabilize the reactive Mn‐intermediates. The closely related complex containing a coordinatively saturated Mn(CO)_4_ fragment (**10**) was also isolated and was found to have a Ru−Mn bond without a supporting/bridging monoazadienyl ligand. However, this complex was found to decompose into monometallic fragments upon exposure to H_2_, rendering it inactive as a hydrogenation catalyst. Despite the mechanistic insights provided by this early heterobimetallic hydrogenation catalyst, the scope of alkene reduction catalyzed by **9** was limited to styrene.

**Scheme 5:**
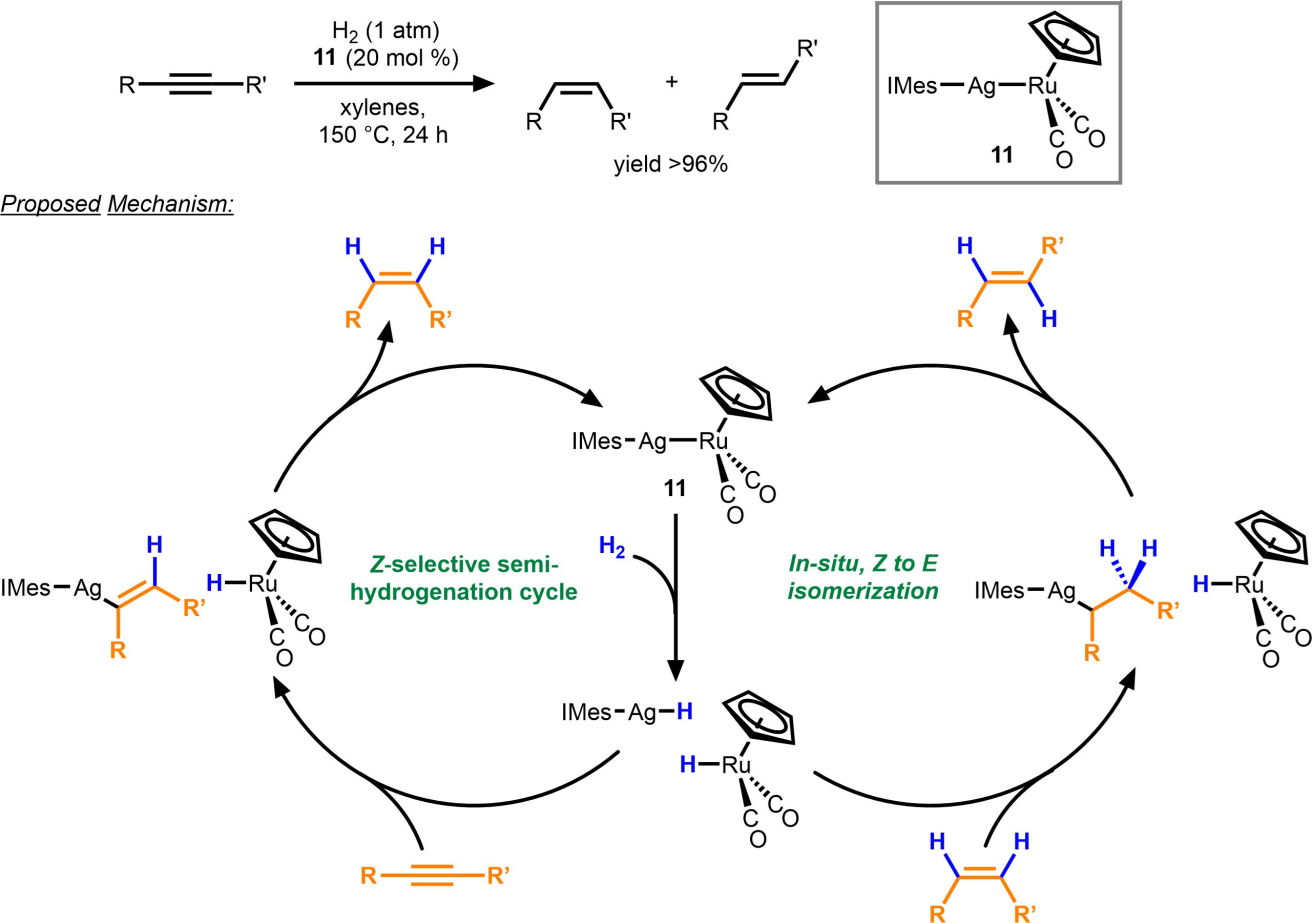
Proposed mechanism for alkyne semi‐hydrogenation catalyzed by Ag/Ru complex **11**.[^32^, ^33^, ^34^]

Scheme 1 and Scheme 4 depict examples of catalysts with metal‐metal bonds, where cleavage into monometallic fragments is detrimental to catalytic activity. However, there are also examples in which dissociation of the bimetallic core into monometallic fragments is crucial to the cooperative bimetallic mechanism. More than two decades after the aforementioned reports, the Mankad group developed heterobimetallic M/M’ (M = Ag, Cu; M’=Fe, Ru) catalysts for *E*‐selective alkyne semi‐hydrogenation with very little propensity for over‐reduction (Scheme 5).^[32]^ Among the bimetallic compounds screened, the Ag/Ru complex **11** shows the highest efficiency and selectivity. Mechanistic investigations revealed that the primary reduction product is the *Z*‐isomer, which is isomerized in situ to the *E*‐alkene in a separate cycle. The identity of the group 8 metal was found to dictate the selectivity; Ru‐based systems were more selective than Fe‐based systems (*E*/*Z*=22.5 and 2, respectively). H_2_ activation was proposed to occur across the M−M bond, cleaving the bimetallic compound into two mononuclear metal‐hydride complexes, followed by the insertion of the alkyne into the group 11 metal‐hydride bond in a *syn*‐fashion. The group 8 metal‐hydride complex then protonates the hydrometallated alkene, re‐forming the M−M bonded active catalyst and the *Z*‐alkene, which is subsequently isomerized to the observed *E*‐product. Diaryl‐substituted internal alkynes were most closely examined, although the terminal alkyne, 1‐ethynyl‐4‐pentylbenzene, could also be reduced. The Ag/Ru system was found to be tolerant towards a variety of functional groups; however, aldehydes were observed to slow down the reaction and invert the selectivity.[^32^, ^33^, ^34^]

**Scheme 6:**
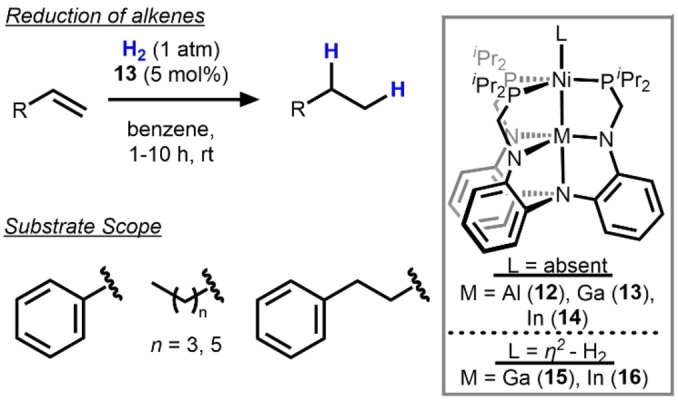
M/Ni (M=Al. Ga, In) bimetallic complexes **12**–**16** and hydrogenation of alkenes catalyzed by Ga/Ni complex **13**.^[35]^

A more recent trend in heterobimetallic hydrogenation catalysis is the combination of a late transition metal with a more Lewis acidic metal center that is either an early metal, a lanthanide, or a Group 13 element. In 2015, the Lu group paired late, low‐valent transition metals with group 13 or lanthanide metalloligands to modulate their activity. The M/Ni complexes (M=Al (**12**), Ga (**13**), In (**14**)), bearing a formal Ni^0^ center were found to catalyze alkene hydrogenation (Scheme 6).^[35]^ Compound **13** was the most competent catalyst for styrene hydrogenation and **12** and **14** demonstrated no activity and very low activity (12 % yield), respectively. The necessity of the bimetallic core was demonstrated by the fact that the monometallic Ni‐only analogue showed <1 % yield for styrene reduction under similar conditions. Both **13** and **14** were observed to bind H_2_ in the apical positions to yield complexes **15** and **16**, respectively. Although **14** was a poor catalyst for alkene hydrogenation, it was observed to efficiently catalyze the isomerization of terminal alkenes to internal alkenes under a H_2_ atmosphere.

**Scheme 7:**
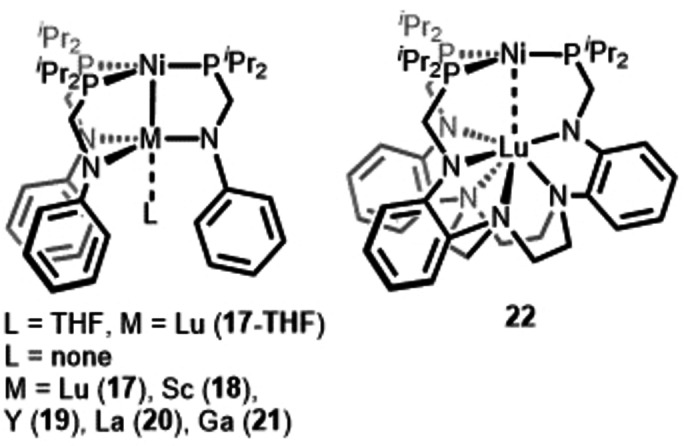
M/Ni complexes examined for alkyne semi‐hydrogenation.^[37]^

This series of heterobimetallic catalysts was extended to 4 f metals by the Lu group using different ligand systems (Scheme 7). The Lu/Ni compounds (**17**, **17‐THF**), which feature three untethered dinucleating amide/phosphine ligands, were also found to catalyze alkene hydrogenation at slightly lower catalytic loading (2.5 mol%) and under more forcing conditions (100 °C, 4 atm H_2_).^[36]^ A marked improvement in reactivity was that **17** catalyzed the reduction of allyl benzene to propyl benzene in near quantitative yield whereas **13** was a poor catalyst for this substrate. Interestingly, complex **22**, which incorporates a 1,4,7‐triazacyclononane (TACN) tether, showed weaker metal‐metal interactions and also performed more poorly as a catalyst for styrene reduction.

Additionally, the complete series of M/Ni compounds (M=Lu (**17**), Sc (**18**), Y (**19**), La (**20**), Ga (**21**)) were systematically investigated as catalysts for *E*‐selective alkyne semi‐hydrogenation.^[37]^ Based on comparative studies conducted with diphenylacetylene as a test substrate, most of the complexes performed competitively (yield of stilbene >90 %) and out‐performed monometallic Ni analogues. Only the Sc/Ni complex **18** showed low conversion. The observed variations in *E*‐selectivity among **17**–**21** were demonstrated to be a consequence of variations in the rate of in situ *Z*‐ to *E*‐ isomerization. The reaction was proposed to proceed via different mechanisms for **21** and complexes **17**, **19**, and **20**. Complex **21** was proposed to first bind H_2_ to form an H_2_ adduct analogous to **15**, followed by reaction with the alkyne to give the reduction product via a hydride‐bridged species. In contrast, complexes **17**, **19**, and **20** were proposed to first bind to the alkyne. The H_2_ bound species, akin to **15** and **16**, were proposed to be the catalytically active species for *Z*→*E* isomerization in all cases.

**Scheme 8:**
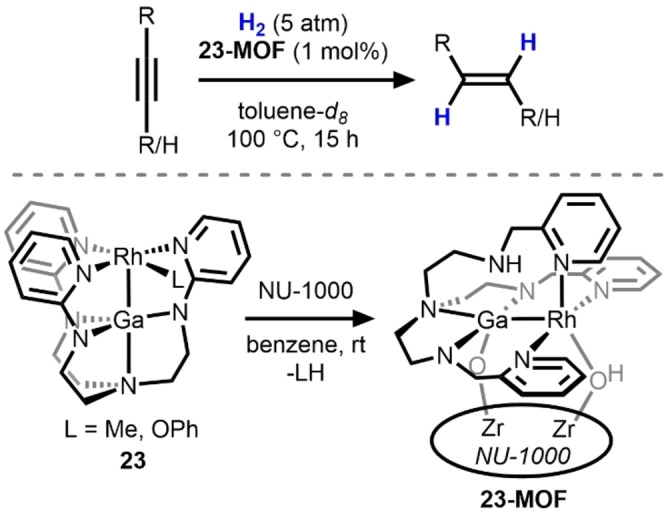
Alkyne semi‐hydrogenation using Rh/Ga catalyst **23** post‐synthetically immobilized in the cavity of a MOF (NU‐1000, **23‐MOF**).^[38]^

Lu's study of bimetallic Ga/M alkyne semi‐hydrogenation catalysts was extended to a metal–organic framework (MOF)‐supported Ga/Rh system, **23‐MOF** (Scheme 8).^[38]^ The Ga/Rh complex (**23**) was immobilized on a NU‐1000 MOF via post‐synthetic modification. The heterobimetallic **23‐MOF** demonstrated a selectivity for the *E*‐alkene and led to very little over‐reduction—catalytic alkene hydrogenation was found to proceed 16 times slower. Similar to previously described examples, the reaction was demonstrated to proceed through a *Z* to *E* isomerization accounting for the observed *E‐*selectivity. The system was also found capable of catalyzing the hydrogenation of terminal alkynes and the substrate scope was broad and included both aryl and alkyl alkynes. The observed chemoselectivity and diastereoselectivity is unique to **23‐MOF** and is not demonstrated by the homogeneous heterobimetallic Ga/Rh complex **23**. Unlike **17**–**21**, **23** was found to degrade upon exposure to H_2_ to form an ill‐defined precipitate that was postulated as a rhodium‐hydride oligomer. It was posited that anchoring **23** to the MOF prevented its decomposition via oligomerization. The monometallic Rh‐only analogue of **23‐MOF** (**Rh‐MOF**) is also an active catalyst; however, **Rh‐MOF** has a high propensity for over‐reduction of the alkyne. A more detailed kinetic analysis of the reaction, along with numerous scrambling experiments, support a mechanism involving two catalytic cycles, reduction to *Z*‐alkene and *Z*‐ to *E*‐isomerization, in tandem proceeding via a common Rh‐hydride species obtained through H_2_ activation.^[39]^

**Scheme 9:**
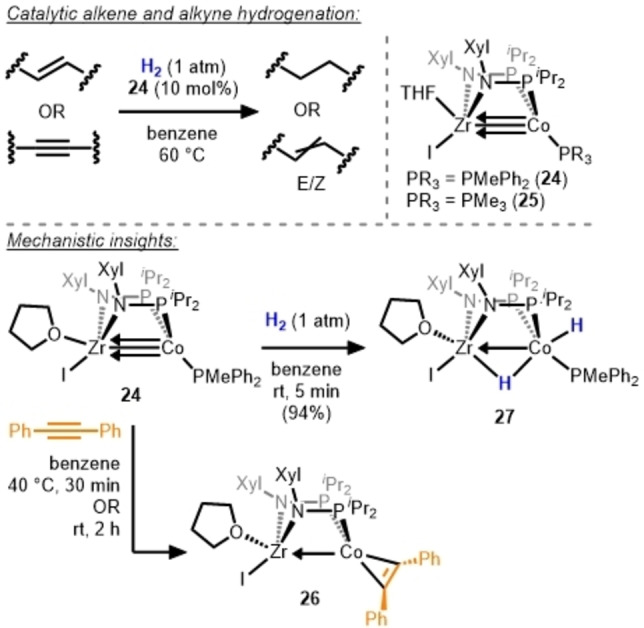
Hydrogenation of unsaturated C−C bonds using Zr/Co heterobimetallic complexes **24**–**25** and stoichiometric reactions of **24** with H_2_ and alkyne.^[40]^

In related work, Thomas and co‐workers employed an early/late heterobimetallic combination to enable hydrogenation catalysis using a first‐row transition metal. The Zr/Co combination with two bridging phosphinoamide ligands was effectively exploited to develop an alkyne semi‐hydrogenation catalyst.^[40]^ In this work, the d^0^ Zr^IV^ center served as a Lewis acidic metallo‐ligand, stabilizing a highly reactive low‐valent Co^−I^ center. The heterobimetallic complexes **24** and **25** (Scheme 9) were found to be efficient catalysts for the hydrogenation of unsaturated alkenes and alkynes. Both terminal and internal alkenes were reduced to alkanes with >90 % yields in 3–48 h at 60 °C and 10 mol% catalyst loading. **24** and **25** are also capable of catalyzing the semi‐hydrogenation of internal alkynes (e. g. diphenylacetylene) with little over‐reduction to alkane. In contrast to previous examples (**11**, **17**–**21**), catalysts **24** and **25** demonstrated poor *E/Z* selectivity because they do not catalyze *Z*→*E* isomerization. The PMePh_2_ derivative, **24**, was found to be significantly more active than the PMe_3_ isomer (**25**) for both alkene and alkyne reduction, implying a dependence on phosphine dissociation.

Stoichiometric reactions between **24** and **25** with H_2_ and substrates led to the formation of new complexes that were also observed under catalytic conditions. Compound **24** underwent a ligand substitution reaction in the presence of diphenylacetylene to yield alkyne adduct **26**; similar reactivity was observed with styrene. Compound **24** also reacts readily with H_2_ to generate dihydride species **27** via oxidative addition of H_2_ across the metal‐metal bond. **26** and **27** were both observed by ^31^P NMR spectroscopy during catalysis. These experimental observations were combined with insight from a computational study by Ke and co‐workers to conclude that H_2_ cleavage, alkyne insertion, and reductive elimination primarily occur at the cobalt center with zirconium playing the important role of stabilizing key intermediates and facilitating reductive elimination via withdrawal of electron density from the low valent Co center.^[41]^ Since catalyst **24** and **25** do not catalyze *Z*→*E* alkene isomerization, the nearly 50/50 *E*/*Z* selectivity must arise from the direct formation of the *E*‐alkene, which could occur via facile Z→*E* isomerization of the cobalt‐bound vinyl intermediate. Indeed, Ke's calculations reveal nearly identical barriers for C−H reductive elimination from the Z and *E* vinyl intermediates, although the mechanism for formation of the *E* vinyl intermediate was not explored.

## Systems with Indirect Metal‐Metal Communication

In the preceding section, the examples discussed included a direct bond/interaction between the metal centers. In contrast, heterobimetallic hydrogenation catalysts are also known where the metals “communicate” or work in tandem, even though there is no direct bond between them. Metal‐metal communication often happens via a conjugated ligand system and is evidenced by the increased reactivity of the heterobimetallic complexes over their monometallic counterparts. Examples of this class of catalysts are discussed in this section.

**Scheme 10:**
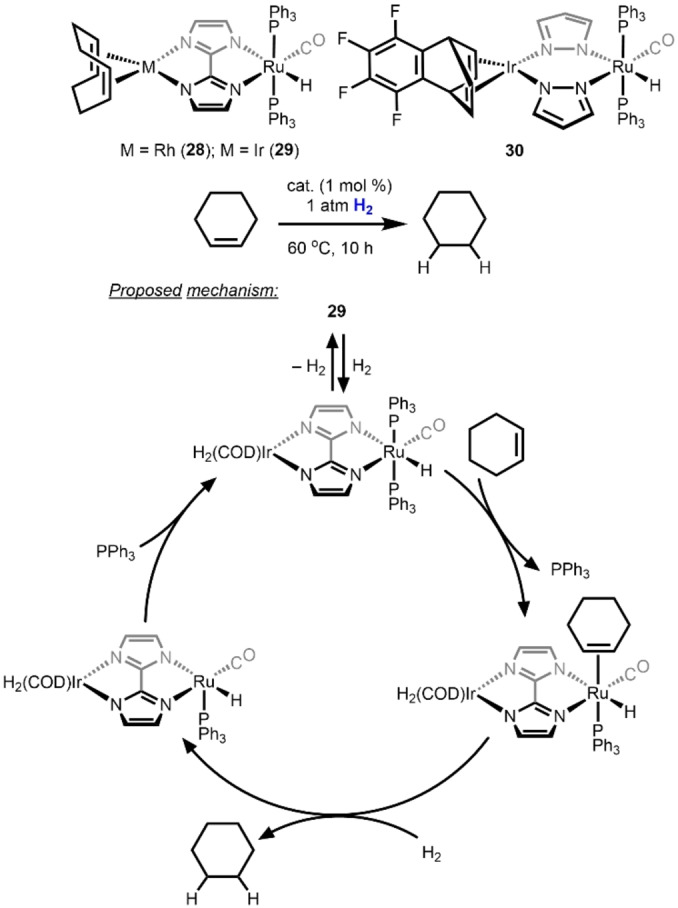
Hydrogenation of cyclohexene catalyzed by Rh/Ru complex **28** and Ir/Ru catalysts **29**–**30** and the proposed hydrogenation mechanism for catalyst **30**.^[43]^

An early report of heterobimetallic hydrogenation catalysts was a series of M/Ru (M=Rh, Ir) complexes bridged by either a 2,2’‐biimidazolate ligand (**28** and **29**) or two pyrazolate ligands (**30**) described by Esteruelas, Oro, and co‐workers beginning in 1988. Compounds **28**–**30** were found to effectively catalyze the hydrogenation of cyclohexene (Scheme 10).[^42^, ^43^] The bimetallic compounds **29** and **30** were found to be significantly more active than their monometallic Rh, Ir, or Ru analogues, prompting the authors to hypothesize electronic communication between the two metals through the conjugated bridging ligand(s). A detailed kinetic investigation of the hydrogenation of cyclohexene catalyzed by **29** supports a catalytic cycle in which alkene binding and hydrogenation occur exclusively at the Ru center, while the appended Rh or Ir center plays the role of tuning electron density through the bridging azolate ligands.^[42]^ The M/Ru 2,2’‐biimidazolate‐ and pyrazolate‐bridged compounds were also reported to be effective catalysts for transfer hydrogenation of cyclohexanone.[^43^, ^44^]

**Scheme 11:**
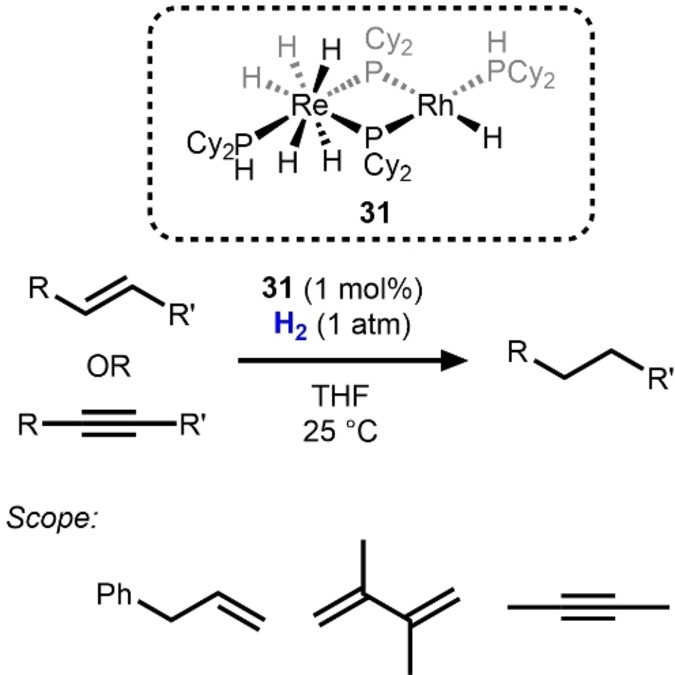
Alkene, diene, and alkyne hydrogenation catalyzed by Re/Rh compound **31**.^[45]^

Another early example of a heterobimetallic hydrogenation catalyst, reported in 1991 by Baker et al., is the Re/Rh compound **31**, which has two dicyclohexylphosphido moieties bridging the two metals (Scheme 11).^[45]^ Complex **31** was generated via addition of H_2_ across both metal centers of (PCy_2_)_2_Re(*μ*−PCy_2_)_2_Rh(COD) and was observed to catalyze the hydrogenation of alkene, diene, and alkyne substrates. In the case of alkynes, complete conversion to alkanes was observed, and no semi‐hydrogenation products were detected during the course of the reaction. While the hydrogenation reactivity observed was comparable to monometallic Rh phosphine hydride compounds, deuterium exchange studies between a deuterated analogue of **31**, **31**‐***d***_**8**_, and allyltrimethylsilane revealed reversible H/D exchange at both the Rh‐hydride, Re‐hydride, and secondary phosphine P−H positions. This deuterium labelling study revealed a high degree of “hydrogen mobility” between the Rh, Re, and P centers in this bimetallic molecule, even if the typical mechanistic steps of alkene hydrogenation steps are occurring exclusively at the Rh site.

**Scheme 12:**
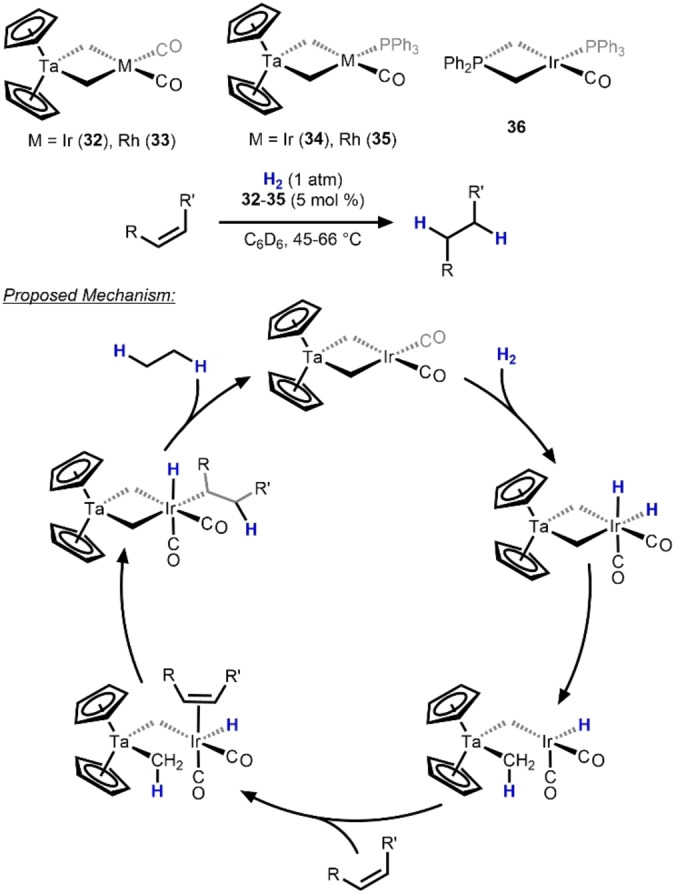
Ta/M (M=Ir, Rh) catalysts **34**–**37** for alkene hydrogenation and a representative monometallic analogue **38**, along with the established bimetallic mechanism for catalytic turnover.[^46^, ^47^, ^48^]

Although most of the examples in this category involve two metals from similar regions of the periodic table, early examples of early/late heterobimetallic hydrogenation catalysts were the Ta/Ir and Ta/Rh complexes **32**–**35** reported by Bergman and co‐workers starting in 1990 (Scheme 12).^[46]^ All four compounds were observed to catalyze the hydrogenation of various alkenes under mild conditions (45–66 °C). Complexes **32** and **34** were found to catalyze the hydrogenation of gaseous alkenes (ethylene, propylene) as well as substituted alkenes (1‐butene, cis‐2‐butene), albeit at a slower rate.^[47]^ Mechanistic investigations using Ta/Ir compounds **32** and **34** revealed the formation of a Ir dihydride intermediate.^[47]^ Furthermore, one of the bridging methylene ligands was observed to participate in the catalytic reaction mechanism via C−H reductive elimination to open an Ir coordination site for alkene binding. Kinetic studies revealed that the rate of deuterium exchange with the bridging methylene was faster than the rate of alkene hydrogenation. Based on these data a mechanism was proposed for the Ta/Ir catalysts that accounts for all empirical observations (Scheme 12). A more recent 2015 computational investigation largely supports the mechanisms proposed in these earlier studies.^[48]^ The corresponding monometallic Ir phosphorus ylide complexes such as **36** were observed to catalytically hydrogenate alkenes 150 times slower than Ta/Ir compounds **32** and **34**. Although the Ta/Rh compound **33** was found to be a faster catalyst for alkene hydrogenation, deuterium exchange studies showed slower kinetics for exchange with the bridging methylene group.^[47]^ Interestingly, although the closely related Ta/Co complex, [Cp_2_Ta(*μ*−CH_2_)_2_CoCp], is known,^[49]^ its ability to activate hydrogen or catalyze hydrogenation reactions has not been reported.

**Scheme 13:**
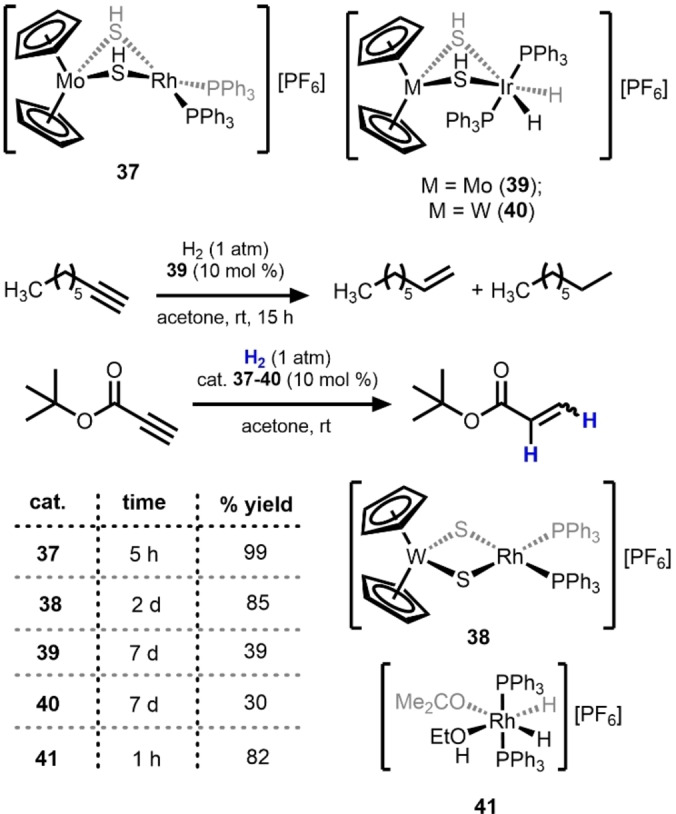
A Group 6/Group 9 heterobimetallic platform for the reduction of terminal alkynes such as 1‐octyne and *tert*‐butyl propiolate.^[50]^

An architecturally similar system with bridging hydrosulfido/sulfido ligands was reported by Hidai in 2002 to catalyze the hydrogenation of alkynes including 1‐octyne and *tert*‐butyl propiolate (Scheme 13).^[50]^ Multiple combinations of Group 6 (Mo, W) and Group 9 (Rh, Ir) metals were tested and the Mo^IV^/Rh^I^ (**37**) combination was found to be the most active catalyst for hydrogenation of both 1‐octyne and *tert*‐butyl propiolate. Unfortunately, **37** was not very selective for the catalytic semi‐hydrogenation of 1‐octyne, affording mixtures of octane (32 %), 1‐octene (22 %), and a mixture of cis and trans isomers of 2‐octene (28 %). In all cases, bimetallic hydrosulfido/sulfido‐bridged complexes were observed in the post‐catalytic reaction mixture, strongly suggesting that the bimetallic system remains intact during catalysis. Although the monomeric Rh analogue [RhH_2_(PPh_3_)_2_(Me_2_CO)(EtOH)][PF_6_] (**41**) showed greater activity than Mo/Rh compound **37** for the hydrogenation of *tert*‐butyl propiolate, a considerable amount of overreduction to the propionate occurs with the monometallic catalyst, demonstrating the potential to control selectivity using a bimetallic approach to attenuate reactivity.

**Scheme 14:**
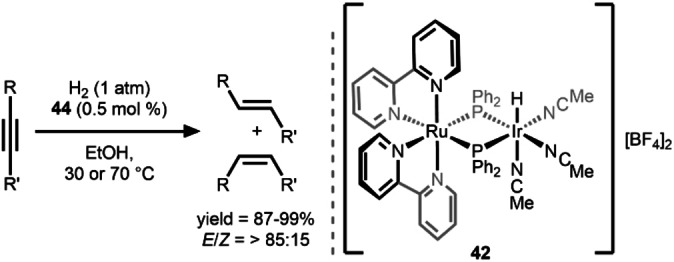
*E*‐selective alkyne semi‐hydrogenation catalyzed by a Ru/Ir system.^[51]^

In a more recent development in the field, Takemoto et al. reported a *d^6^/d* ^*6*^ Ru/Ir complex **42** in 2019 and described its ability to efficiently catalyze the *E*‐selective semi‐hydrogenation of alkynes (Scheme 14).^[51]^ The catalyst is broadly effective with alkynes with at least one phenyl/aryl group on the alkyne, with >99 % yield and *E*/*Z* >90 for substrates without reactive functional groups. Furthermore, **42** was shown to catalyze semi‐hydrogenation of the internal dialkyl alkene, 4‐octyne (yield >99 %), albeit with slightly hampered selectivity (*E*/*Z*=85/15). In contrast to the previous example **40**, **42** out‐performed monometallic Ru and Ir analogues bearing similar ligand environments, illustrating the necessity of the bimetallic framework for realizing the observed reactivity. Compound **42** efficiently catalyzed the isomerization of *Z*‐stilbene to *E*‐stilbene under catalytically relevant conditions, indicating that the observed *E*‐selectivity stems from an in situ *Z*→*E* isomerization following the catalytic reduction to the *Z*‐alkene, in line with previously described heterobimetallic catalysts (i. e. **11**, **17**–**21**, **23‐MOF**).

**Scheme 15:**
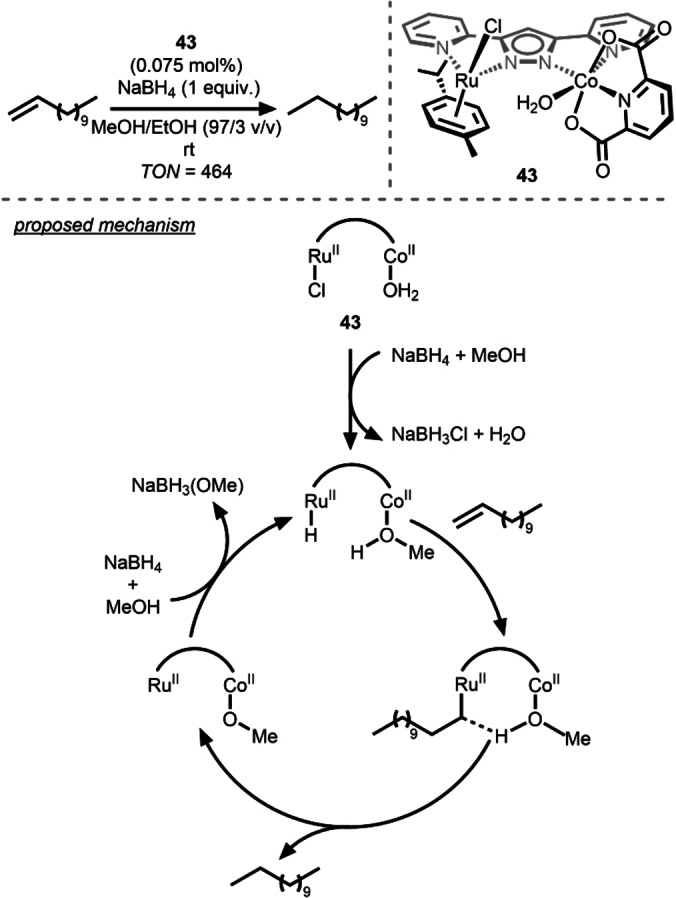
Reduction of 1‐dodecene using a Ru/Co catalyst.^[52]^

Many contemporary research efforts on bimetallic catalysis involve carefully designed dinucleating ligands to link the two metals in close proximity. An example of this approach in the heterobimetallic regime is the pyrazolyl‐linked Ru/Co compound reported by Hong and co‐workers in 2019. The Ru^II^/Co^II^ complex **43** was constructed by linking two distinct metal fragments using a dinucleating dipyridylpyrazolyl ligand. Compound **43** was found to catalyze the hydrogenation of 1‐dodecene to *n*‐dodecane at room temperature using NaBH_4_ with >50 % conversion to *n*‐dodecane (Scheme 15).^[52]^ The Ru⋅⋅⋅⋅Co distance in **43** was found to be 4.357(1) Å, far too long for a direct metal‐metal bond or interaction. However, the importance of the bimetallic architecture is evident from the lack of catalytic activity of monometallic Ru^II^ and Co^II^ analogues with similar coordination environments either individually or as an equimolar mixture. Although up to 464 turnovers could be achieved using **43**, no catalytic activity was observed under an H_2_ atmosphere in the absence of NaBH_4_ and MeOH was shown to be crucial for catalysis. On the basis of stoichiometric reactions and kinetic investigations, the authors proposed a catalytic cycle involving alkene insertion into a Ru^II^−H bond and protonolysis of the resulting Ru−alkyl bond by a Co‐bound molecule of MeOH to release the alkane product. Although this system does not perform hydrogenation directly with H_2_, it provides an excellent example of a cooperative mechanism directly involving both metals.

Although the focus of this minireview has been transition metal heterobimetallic catalysts, any work looking at heterobimetallic catalysts would be incomplete without mentioning several examples of elegant main group heterobimetallic catalysts utilizing combinations of alkali or alkaline earth metals and group 13 metals/metalloids. In these main group examples, ligands impart no ligand‐field stabilization, as is the case of transition metals. Therefore, these systems employ far more straightforward ligand systems and rely on strong ionic interactions and steric factors to regulate catalytic activity. Some relevant examples are discussed here.

**Scheme 16:**
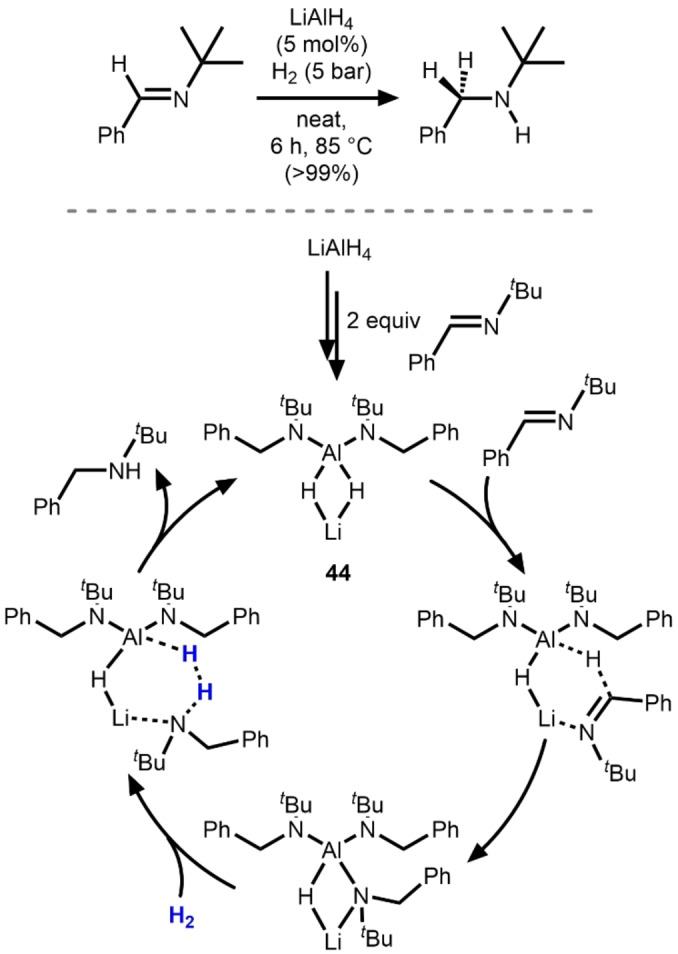
Proposed mechanism for imine reduction using LiAlH_4_ as catalyst.^[53]^

In 2018, Harder and co‐workers reported that the reduction of imines only requires catalytic LiAlH_4_ when performed under an H_2_ atmosphere (Scheme 16).^[53]^ Upon screening various conditions, the authors found that the loading of LiAlH_4_ could be reduced as low as 2.5 mol% while still achieving >99 % conversion under relatively mild conditions (1–7 bar H_2_, 85 °C, solvent‐free). Stoichiometric reactions were combined with DFT studies to propose a cooperative bimetallic mechanism (Scheme 16).^[54]^ The reaction was found to be far less efficient with NaAlH_4_ or KAlH_4_ and did not proceed at all with [^*n*^Bu_4_N][AlH_4_], hinting at the importance of the alkali metal cation in the catalytic cycle. Moreover, replacing the Al in LiAlH_4_ with another Group 13 metal (B or Ga) shut down catalytic activity. The reaction of LiAlH_4_ with 2 equiv. of imine at 80 °C led to the isolation of the bis(amido) aluminium complex **44**, which is proposed to be the actual catalyst in the catalytic cycle. Insertion of a third imine into one of the remaining hydrides is proposed to be aided by coordination of the imine functionality to the Lewis acidic Li^+^ cation. The Li^+^ cation is, however, not involved in the addition of H_2_ to the resulting tris(amide) intermediate and hydrogenolysis of the Al−N bond occurs exclusively at the Al center. In a related report from the same group, alkaline earth aluminates M(AlH_4_)_2_ (M=Mg, Ca, Sr) were examined and shown to be both more efficient and more broadly applicable than LiAlH_4_ for imine hydrogenation.^[55]^ The catalytic activity of M(AlH_4_)_2_ was found to be dependent on the size of the *s* block metal, but in this case the larger alkaline earth cation (Ca^2+^, Sr^2+^) led to more active catalysts than the smaller Mg^2+^ ion. A cooperative heterobimetallic mechanism similar to that outlined in Scheme 16 and was proposed.

## Conclusions

As can be seen from the examples in this minireview, heterobimetallic complexes have been investigated as hydrogenation catalysts for more than 35 years. Although there was limited success at the inception of this research area, there has been much progress in this field in the past 10 years. Heterobimetallic complexes have been particularly successful as catalysts for alkene and alkyne hydrogenation, especially for semi‐hydrogenation of alkynes enabled due to the chemoselectivity demonstrated by these complexes. In summary, building upon historical precedents, recent developments have demonstrated that heterobimetallic hydrogenation catalysts can lead to more active and selective catalysts than their monometallic constituents and homobimetallic analogues.

Despite these advances, there remains substantial room for improvement and advances in the field of catalytic hydrogenation by heterobimetallic compounds. For example, the primary application of homogeneous hydrogenation reactions in the pharmaceutical industry is asymmetric hydrogenations (e. g. Noyori‐type catalysts), but there are currently no documented examples of heterobimetallic catalysts for asymmetric hydrogenations. This unexplored area of research is perhaps even more intriguing considering the unique chemoselectivity offered by heterobimetallic hydrogenation catalysts. Mechanistic insight into cooperative bimetallic mechanisms is crucial for the design of such asymmetric bimetallic catalysts in order to understand which metal site will play the most important role in controlling enantioselectivity and, therefore, where to place chiral functional groups within the bimetallic framework. Another area where heterobimetallic catalysts may find utility is in the reduction of commodity chemicals. For instance, although examples of polymer reduction and hydrogenative lignin valorization are known with monometallic and homo‐bimetallic catalysts, there are currently no documented heterobimetallic catalysts for these applications. Lastly, even in absence of practical applications for heterobimetallic homogeneous catalysts in scalable industrial processes, the fundamental insights provided by the study of such molecular compounds undoubtedly will continue to provide fundamental information of relevance to the mechanisms at play in heterogeneous bimetallic hydrogenation catalysts.

## Conflict of Interests

The authors declare no conflict of interest.

1

## Biographical Information

*Preshit Abhyankar received his PhD from the University at Buffalo under the supervision of David C. Lacy. His graduate work was focused on the exploration of dinucear Mn(I)‐complexes for their application in H_2_ activation and catalytic hydrogenation. Preshit then moved to The Ohio State University as a postdoctoral scholar with Prof. Christine Thomas where he investigated early/late heterobimetallic complexes for catalytic applications*.

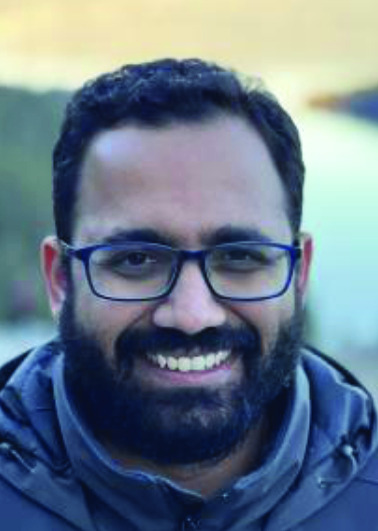

## Biographical Information

*Christine Thomas is a Professor at The Ohio State University and a Fellow of the Royal Society of Chemistry. She received her Ph.  D. in 2006 at Caltech and pursued postdoctoral work at Texas A&M University. After beginning her career at Brandeis University in 2008, she moved to The Ohio State University in 2018. She has developed a research program focused on the development of novel coordination compounds, including heterobimetallic complexes, with applications in organometallic catalysis and small molecule activation*.

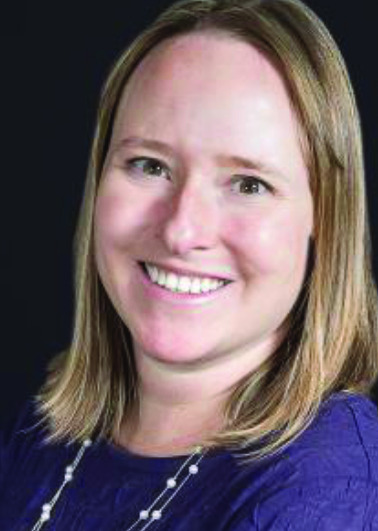

## Data Availability

Data sharing is not applicable to this article as no new data were created or analyzed in this study.
